# New Method for a SEM-Based Characterization of Helical-Fiber Nonwovens

**DOI:** 10.3390/polym14163370

**Published:** 2022-08-18

**Authors:** Ying Li, Guixin Cui, Yongchun Zeng

**Affiliations:** 1 College of Textiles, Donghua University, Shanghai 201620, China; 2 China Textile Academy, Jiangnan Branch, Shaoxing 312071, China

**Keywords:** characterization, helical, nonwoven, curvature

## Abstract

The lack of tools particularly designed for the quantification of the fiber morphology in nonwovens, especially the multi-level structured fibers, is the main reason for the limited research studies on the establishment of realistic nonwoven structure. In this study, two polymers, cellulose acetate (CA) and thermoplastic polyurethane (TPU), which have different molecular flexibility, were chosen to produce nonwovens with helical nanofibers. Focusing on the nonwovens with helical fibers, a soft package was developed to characterize fiber morphologies, including fiber orientation, helix diameter, and curvature of helix. The novelty of this study is the proposal of a method for the characterization of nanofibrous nonwovens with special fiber shape (helical fibers) which can be used for curve fibers. The characterization results for the helical-fiber nonwoven sample and the nonwoven sample with straight fibers were compared and analyzed.

## 1. Introduction

Nature’s creatures have been endowed with a tremendous number of excellent biological materials with fascinating structures, which exhibit numerous optimized functions [1,2,3]. Helical structure is one of them. Helical micro/nanofibers, which combine a helical structure with micro/nanofibers, have attracted increasing interest due to their unique structure and characteristics [4,5,6,7]. Electrospinning is a versatile technique to produce fibers with a diameter ranging from nanometers to several micrometers effectively. Electrospun micro/nanoproducts have shown their potential in the sorption and reuse of crude oil, tissue engineering, artificial muscles, and other smart systems [8,9,10]. The traditional setup for electrospinning uses a single needle, the need of complex nanostructure-based advanced functional nanomaterials has promoted the appearance of several kinds of multifluid electrospinning processes, such as tri-axial electrospinning [11], tri-fluid electrospinning [12], coaxial electrospinning [13] with a side-by-side core, and co-electrospinning. Micro/nanofibrous nonwovens that contain helical fibers (called “helical-fiber nonwovens”), which can be produced via electrospinning, have the advantage of large superficial area, large porosity, and good mechanical properties [12,14,15,16,17]. However, one major challenge in the research is the lack of quantitative analysis of helical fiber morphology. Image processing technique, which is a powerful tool to acquire information from images, has been utilized to characterize and analyze nonwovens since the 1990s [18,19,20]. Recently, Moll et al. measured fiber orientation of fiber injection molded nonwovens by image analysis [21].

In this work, two polymers, cellulose acetate (CA) and thermoplastic polyurethane (TPU), which have different molecular flexibility, were chosen to produce nonwovens with helical nanofibers. The objective of this work was to provide an efficient software package for characterizing fiber morphology in helical-fiber nonwovens, including fiber diameter, fiber orientation, as well as helical diameter and curvature. The presence of the characterization for the helical-fiber nonwovens can lead to modelling of the nonwovens’ behaviors. A new algorithm was produced to quantify helical diameter and curvature of helical fibers. This image analysis was achieved by application of MATLAB. 

In the following sections, the image acquisition, implementation details, and analysis procedures are proposed.

## 2. Experiments

### 2.1. Materials and Solution Preparation

Thermoplastic polyurethane (TPU; Desmopan DP 2590A) was purchased from the Bayer Materials Science, Leverkusen, Germany. Cellulose acetate powders (CA; 39.8 wt% acetyl, molecular of mass of Mn ~ 30,000). N,N-dimethylformamide (DMF; 0.944 g/mL) and N,N-dimethylacetamide (DMAc; 0.937 g/mL at 25 °C) were purchased from Sigma-Aldrich, St. Louis, MO, USA. Acetone was purchased from Shanghai Lingfeng Chemical Reagent Co. Ltd., Shanghai, China. Lithium chloride anhydrous (LiCl, Mw = 42.39 g/mol) was provided by the Shanghai Chemical Reagents, Shanghai, China. All of these materials were directly used without any further purification.

TPU was dissolved in DMF with different concentrations of 12 wt% and 14 wt%. CA solution of 17 wt% was prepared by dissolving CA powder in a mixture of DMAc/acetone (1:2) with 2 wt% LiCl. CA solution of 15 wt% was prepared by dissolving CA powders in DMAc. All of these solutions were stirred for 8 h at room temperature.

### 2.2. Co-Electrospinning

**Figure 1:**
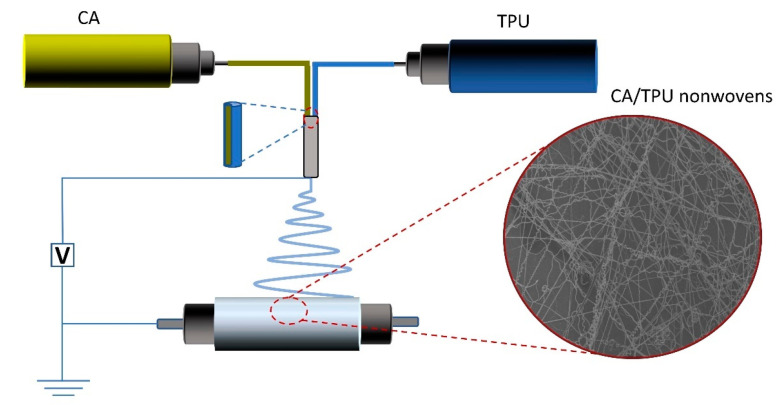
The schematic of co-electrospinning system and the SEM image of CA/TPU nonwovens.

The co-electrospinning system used in this study is shown in Figure 1. The system is an off-centered core-shell spinneret in which the inner needle is eccentrically situated inside the outer one. This study focused on the characterization of helical nanofibers in nonwovens, according to our previous study [22]—we used the optimized processing conditions to obtain the target nonwoven sample. The helical-fiber nonwoven sample was electrospun by CA (15 wt%)/TPU (12 wt%), while the nonwoven sample with straight fibers was electrospun by CA (17 wt%)/TPU (14 wt%). A voltage of 20 kV was applied to the co-electrospinning. The distance from the spinneret to the collector was 15 cm. In this research, all the experiments were performed at ambient temperature of about 25 °C and relative humidity of 40–60%.

## 3. Image Processing-Methodology

### 3.1. Image Acquisition

**Figure 2:**
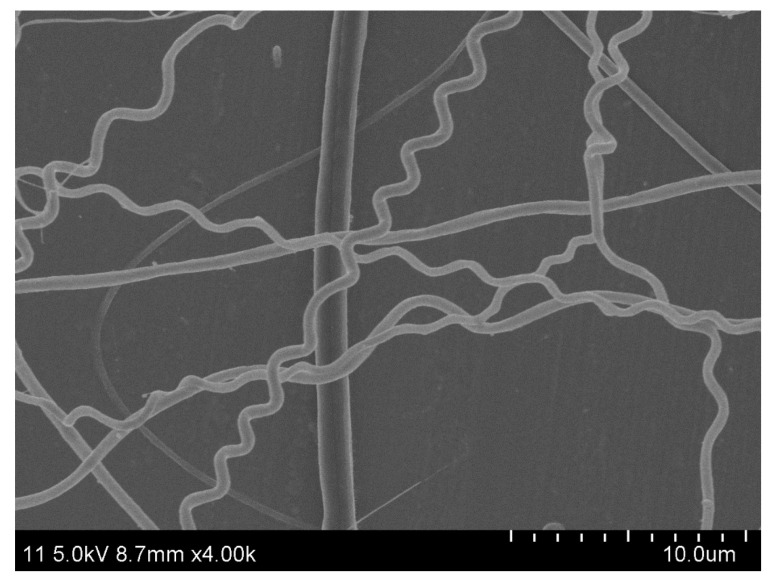
The SEM image of the helical-fiber nonwoven sample.

Figure 2 shows the image of the obtained helical-fiber nonwoven sample. The field emission scanning electron microscope (FE-SEM, SU8010, HITACHI, Tokyo, Japan, 5 kV, 10 mA, 8 mm working distance) was used to create the digital image of the nonwoven sample. In digital images, the geometric information of fibers is measured by pixels. The format of the image acquired by SEM was classified as the grayscale image, where each pixel was represented by a value from 0 to 255. A zoom factor (i.e., the real length of one pixel), which is determined from the SEM image (Figure 2), was used to transform the pixel to geometrical real size. In this case, each pixel had a dimension of 0.0246 μm in length and width.

### 3.2. Image Vectorization

The digital image acquired by SEM was read and saved as a matrix of discrete pixels. An image vectorization progress, which involves image binarization, connected-component labeling, and boundary extraction, was implemented to transform the digital image to a vector graph and to extract the fiber geometry consequently.

The m×n pixel matrix can be expressed by a discrete function f(i,j) in the Cartesian coordinate system:

(1) $$f\left(i,j\right)=\left[\begin{array}{ccc} \begin{array}{cc} f\left(1,1\right) & f\left(1,2\right)\\ f\left(2,1\right) & f\left(2,2\right) \end{array} & \cdots & \begin{array}{c} f\left(1,m\right)\\ f\left(2,m\right) \end{array}\\ \vdots & \ddots & \vdots \\ \begin{array}{cc} f\left(n,1\right) & f\left(n,2\right) \end{array} & \cdots & f\left(n,m\right) \end{array}\right],\;i=1,\;2,\;\ldots ,n,\;j=1,\;2,\ldots ,m.$$

**Figure 3:**
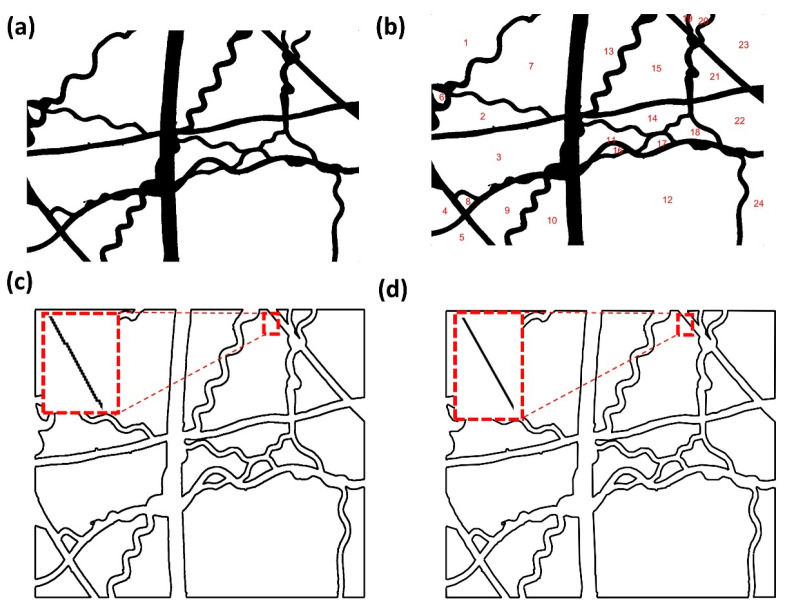
Vectorization of the helical-fiber nonwoven sample: (**a**) binarization image; (**b**) labelled binarization image; (**c**) boundaries extraction without refinement; (**d**) boundaries extraction after refinement.

To binarize the digital image, a global threshold was determined according to Otsu’s [23] method and turned out to be 109 (0.4275 × 255) in this case. All the pixel values in f(i,j) were set to two values, 1 (white) or 0 (black). The pixel values above 109 were set as 1, and the other ones were set as 0. The final binary image is shown in Figure 3a. 

We used boundary of pores to obtain the geometric information of fibers. Consequently, the binary image (Figure 3a) was segmented into a series of discrete pore domains. To label the pore domains, a connected-component labeling algorithm [24] was adopted. The pore domain, whose pixel value is 1 (white), was labeled and recorded by searching the eight neighboring domains until no white pixel was found. The total labeled pore domains form a label matrix. Regionprops function was applied to sort the label matrix into different label sets to isolate individual pore domains. Figure 3b shows the labeled binary image. It can be seen that the fibers are distinguished from the pores.

Followed by the labeling procedure, boundaries of pores were extracted and their coordinates registered using the boundary extraction algorithm. The vector graph, which is constituted of the profiles of the boundaries, is shown in Figure 3c. It can be seen that the boundaries present as jagged polylines containing huge numbers of points (i.e., pixel positions). We used the method proposed by Prasad [25,26] to make dominant point detection and refine the boundaries based on the dominant points. Figure 3d shows the pore boundaries after refinement. It can be seen that the boundaries are smoothed under the condition where their curvature properties are retained. 

### 3.3. Fiber Diameter, Length, and Orientation

The fiber geometric characteristics, including fiber morphology (fiber diameter, fiber orientation, fiber length) and helix geometry (helix radius, helix pitch), were characterized for the nonwoven model generation. 

**Figure 4:**
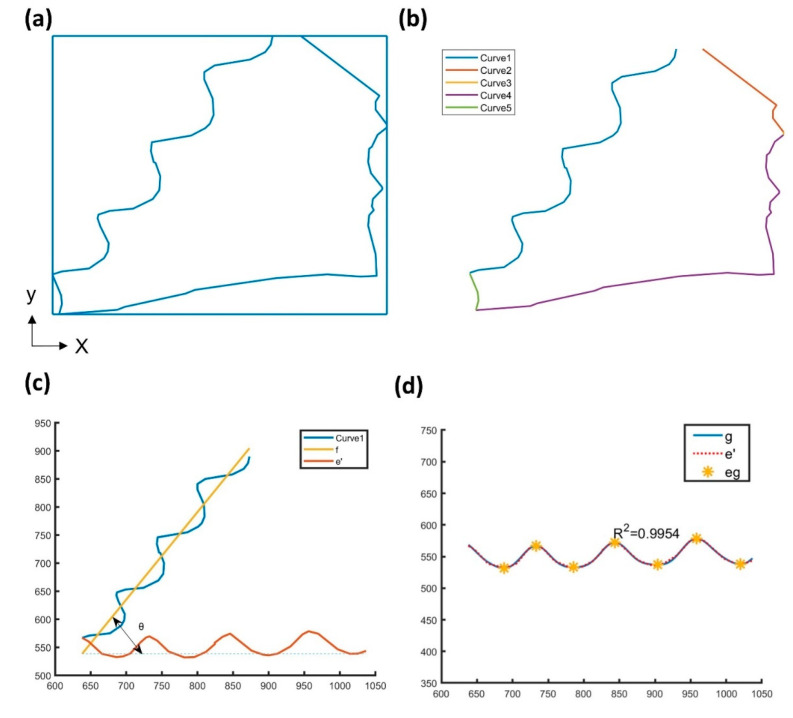
The characterization of fibers geometries by analyzing the helical-fibers of nonwoven sample: (**a**) edge detection; (**b**) dominant points edge detection; (**c**) the original Curve 1 and the rotated curve e’; (**d**) the Fourier fit curve and extreme points eg.

The average fiber diameter was analyzed from the labeled binary image (Figure 3b) and is given by D=S/L, where S is the total area occupied by the fibers, and L is the total length of the fibers. S can be determined by the number of the black pixels and the unit dimension of each pixel, and L is the pixel number of the boundary between black and white pixels. Fiber length, fiber orientation, and helix geometry were analyzed from the vector graphs. Figure 4a shows an individual pore domain extracted from the vector graph. A bounding rectangle of the pore domain was determined by searching the nearest and the farthest points in the x and y directions. Then the boundary was divided into five curves (Figure 4b) based on the intersecting points between the boundary and the bounding rectangle. As mentioned above, pores in the nonwoven sample are formed by interlacing fibers. Therefore, the pore boundary in Figure 4a is simultaneously the boundary of the constituent fibers. The curves possess the information of fiber length, fiber orientation, and helix geometry of the helical fiber. In the following, Curve 1 was chosen as a fiber segment to characterize the fiber geometry.

Curve 1 can be expressed as e = {P1 P2 … PN}, where Pi (*x_i_, y_i_*) is the $i_{th}$ point in the Cartesian coordinate system, and *N* is the number of pixel positions in the curve. *l* is given by

(2) $$l=\sum_{i}^{N}{\left[{\left(x_{i}-x_{i-1}\right)}^{2}+{\left(y_{i}-y_{i-1}\right)}^{2}\right]}^{\frac{1}{2}}$$

The inclination of the best-fit line of Curve 1 serves as the orientation of the fiber segment in the x-y plane. Curve 1 and its fit line (f), which is derived using the least-square error method, is shown in Figure 4c. The angle *θ*, which represents the inclination angle of M relative to the x-direction, can be derived from the slope a of M.

(3) $$\begin{array}{c} a=\frac{\sum_{i=1}^{N}x_{i}y_{i}-\frac{1}{N}\sum_{i=1}^{N}x\sum_{i=1}^{N}y}{\sum_{i=1}^{N}x_{i}^{2}-\frac{1}{N}{\left(\sum_{i=1}^{N}x_{i}\right)}^{2}} \end{array}$$

(4) θ=arctana 

where *N* is the number of points in the curve, and ($x_{i}$,$y_{i}$) is the coordinate of the $i_{th}$ point. 

### 3.4. Helix Diameter and Curvature

The curves shown in the vector graph represent the projection of helical fibers in the x-y plane. To characterize helix geometry of the helical fiber, the Fourier series with sine-cosine form was used to describe the curve due to the period characteristic of a helix. To start with, to eliminate the influence of fiber orientation on curve fitting, Curve 1 (e) rotates −θ around P1 to the x-direction and becomes e’ = {P1 P2‘ … PN’}. Figure 4c shows the curves e’ and the Fourier fit curve is given by the expression

(5) $$f\left(x\right)=a_{0}+\sum_{1}^{nc}\left[a_{n}\cos\left(n\omega x\right)+b_{n}\sin\left(n\omega x\right)\right]$$

The number of Fourier coefficients employed in the present calculation is taken as *nc* = 8, leading to a 0.9954 R^2^ value. This correlation coefficient (close to 1) provides the reliability of the curve fit. The parameters a_0_ (the distance of the axial line of e’ away from the x-axial), w (the minimum frequency of the trigonometric functions), Fourier coefficients an and bn are as follows:

a_0_ = 551.3 ω = 0.01437

a_1_ = 0.5819 b_1_ = 5.045      a_2_ = −0.6606 b_2_ = −0.7522   a_3_ = −1.465 b_3_ = −2.75       a_4_ = −0.4519 b_4_ = −18.24     a_5_ = −0.3821 b_5_ = 2.303         a_6_ = −0.166 b_6_ = −0.02012         a_7_ = −0.8761 b_7_ = −0.1932   a_8_ = −2.499 b_8_ = 1.339

As the parameter x varies from P1 to PN’, the Fourier fit curve g is shown in Figure 4d.

Helix geometry can be characterized by the pitch, radius, and curvature in terms of pitch and radius. The helix pitch (*p*) is determined by the x-distance of the adjacent extreme points of g (Figure 4d), while the helix diameter (*d_h_*) is determined by the y-distance of the extreme points of g. The corresponding helix curvature is expressed by

(6) $$\kappa =\frac{\frac{d_{h}}{2}}{\left({\left(\frac{d_{h}}{2}\right)}^{2}+{\left(\frac{p}{2*\pi }\right)}^{2}\right)}\;$$

## 4. Characterization Results

**Figure 5:**
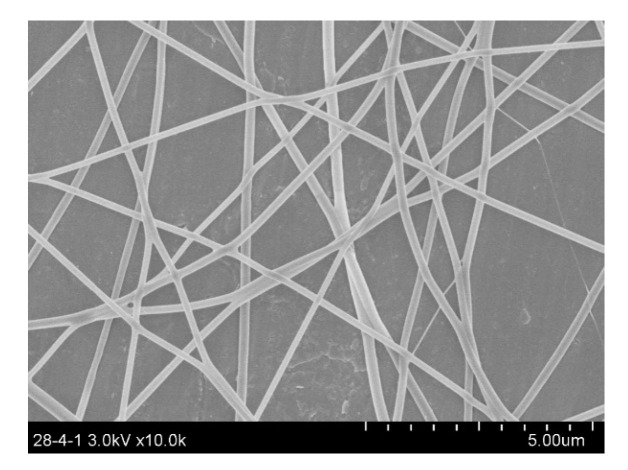
The SEM image of the nonwoven sample with straight fibers.

**Figure 6:**
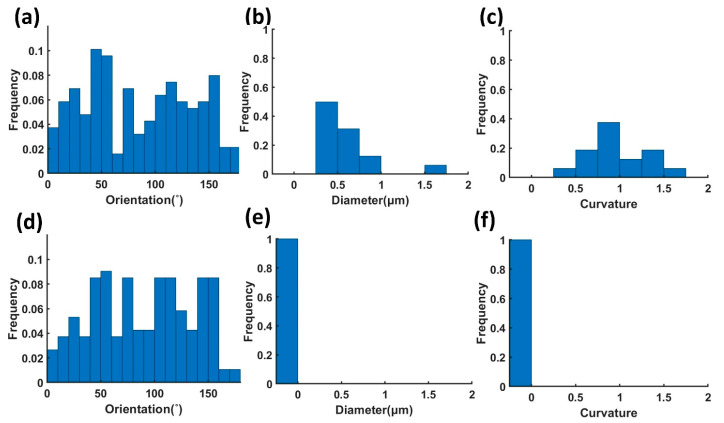
Comparison of characterization results for (**a**–**c**) helical-fiber nonwoven sample and (**d**–**f**) nonwoven sample with straight fiber, where (**a**,**d**) are fiber orientation distributions; (**b**,**e**) are helix diameter distributions; (**c**,**f**) are curvatures of helix distributions.

According to the developed soft package, the characterization based on the images of two types of nonwoven samples were carried out. Figure 2 and Figure 5 show the helical-fiber nonwoven sample and the nonwoven sample with straight fibers, respectively. Figure 6 shows the comparison of characterization of fiber orientation, helix diameter, and helix curvature for the two types of nonwoven samples.

To verify the characterization of fiber diameter, which is 0.6 μm for the helical-fiber nonwoven sample and 0.22 μm for the sample with straight fibers, DiameterJ plugin [27], which is an open source for nanofiber diameter measurement tool, was used to analyze the two samples. The results (0.57 μm for the helical-fiber sample and 0.19 μm for the sample with straight fibers) turn out to be in accordance with the results using our method. 

Figure 6a,d shows that the orientation distributions of fibers for the two samples have similar characteristic and present the anisotropy of the nonwovens. Figure 6b,e shows the helix diameter distributions of fibers for the two cases. The fiber helix diameters of the helical-fiber nonwoven sample are in the range of 0.25–1.5 μm, with the average diameter of 0.6 μm. As expected, the helix diameter frequency of the sample with straight fibers is 0. 

Figure 6c,f shows the curvatures of helixes of the fibers for the two cases. For the helical-fiber nonwoven sample, the curvature of the helical fibers ranges from 0.25–1.75 μm^−1^, with most of the curvatures concentrated at 0.5–1.5 μm^−1^. As expected, the frequency of fibers processing curvature for the nonwoven sample with straight fibers is 0.

## 5. Conclusions

The morphology of fibers in non-woven fabrics is essential for fabric performance. However, the lack of tools for characterization of the helix microstructure observed in SEM micrographs makes it difficult to quantify the morphological parameters of helical-fiber nonwovens. This study proposes a method for the characterization of helical fibers in nonwovens. By developing a new method to extract the fiber morphology from scanning electron microscope images, the fiber orientation, helix diameter, and curvature of the helix were characterized. The software package provides a user-friendly code for analyzing helical fiber morphologies of digital images obtained by scanning electron microscope images. This software package can encourage other researchers to investigate micromechanical behavior using such numerical methods. 

## Data Availability

The data presented in this study are available on request from the corresponding author.
